# Diabetic peripheral neuropathy based on Schwann cell injury: mechanisms of cell death regulation and therapeutic perspectives

**DOI:** 10.3389/fendo.2024.1427679

**Published:** 2024-08-12

**Authors:** Lijiao Wu, Xiang Jin Wang, Xi Luo, Jingqi Zhang, Xinyi Zhao, Qiu Chen

**Affiliations:** ^1^ Department of Endocrinology, Hospital of Chengdu University of Traditional Chinese Medicine, Chengdu, China; ^2^ School of Clinical Medicine, Chengdu University of Traditional Chinese Medicine, Chengdu, China; ^3^ School of Sports Medicine and Health, Chengdu Sports University, Chengdu, China; ^4^ Jiangsu Key Laboratory for Functional Substance of Chinese Medicine, School of Pharmacy, Nanjing University of Chinese Medicine, Nanjing, China; ^5^ College of lntegrated Traditional Chinese and Western Medicine, Hunan University of Chinese Medicine, Hunan, China

**Keywords:** diabetic peripheral neuropathy, Schwann cells, hyperglycemia, programmed cell death regulation, neuropathic pain (NP)

## Abstract

Diabetic peripheral neuropathy (DPN) is a complication of diabetes mellitus that lacks specific treatment, its high prevalence and disabling neuropathic pain greatly affects patients’ physical and mental health. Schwann cells (SCs) are the major glial cells of the peripheral nervous system, which play an important role in various inflammatory and metabolic neuropathies by providing nutritional support, wrapping axons and promoting repair and regeneration. Increasingly, high glucose (HG) has been found to promote the progression of DPN pathogenesis by targeting SCs death regulation, thus revealing the specific molecular process of programmed cell death (PCD) in which SCs are disrupted is an important link to gain insight into the pathogenesis of DPN. This paper is the first to review the recent progress of HG studies on apoptosis, autophagy, pyroptosis, ferroptosis and necroptosis pathways in SCs, and points out the crosstalk between various PCDs and the related therapeutic perspectives, with the aim of providing new perspectives for a deeper understanding of the mechanisms of DPN and the exploration of effective therapeutic targets.

## Introduction

Diabetes, a serious chronic disease, is growing worldwide and is now one of the top ten leading causes of death worldwide (1). Worldwide, an estimated 537 million adults between the ages of 20 and 79 have diabetes, and the number is expected to increase to 643 million by 2030 (2). Persistent hyperglycemia, dyslipidemia, and insulin deficiency or dysfunction contribute to the multiple chronic complications of diabetes mellitus, and the vascular and neuronal systems, as providers of nutrients to all organs as well as connectors, play an important role in the development of diabetes mellitus complications (3). Diabetic peripheral neuropathy (DPN) as the most common complication of diabetes mellitus (4), is a debilitating and complex neurologic condition that has gained widespread attention due to its prevalence of up to 50% in patients with type 1 and type 2 diabetes mellitus (5). It affects patients’ quality of life by causing numbness, neuropathic pain, and severe lower extremity amputations (6), more studies have found that diabetic autonomic neuropathy increases the risk of death by more than twofold (7). Despite the numerous studies on the treatment of DPN, current treatments are limited to improved glycemic control, lifestyle and dietary interventions, and pain management, it remains the only diabetic microangiopathy that lacks specific therapy (8). Therefore, an in-depth exploration of the specific pathogenesis of DPN is essential to facilitate the prevention and treatment of the disease. Further studies have revealed that cellular therapies may be an effective way to address the underlying causes of neuropathy, and cellular therapies for both MSC and pulp stem cell transplantation point to the realization of a neurorestorative effect through the protection of Schwann cells (SCs) (9, 10), At this juncture, studies have substantiated that human tonsil-derived mesenchymal stem cells (MSCs) that have undergone differentiation into Schwann cells (TMSC-SCs) have the capacity to facilitate nerve regeneration in animal models of peripheral nerve injury. Additionally, Schwann cell-derived exosomes (SCEVs) have been demonstrated to possess therapeutic potential for the management of DPN (11, 12), the results of these studies indicate that SCs may play a significant role in the pathogenesis of peripheral neuropathy.

SCs, as the main glial cells in the peripheral nervous system, possess regenerative properties of interest and have important roles in the nervous system in coordinating the gradual formation of peripheral nerves, ensuring neuronal survival, axonal support and myelination, and promoting neurodegeneration and regeneration (13, 14). In addition, SCs can influence neuronal responses to stimuli, respond rapidly in the face of injury and coordinate intraneural changes, playing a key role in the development and maintenance of neuropathic pain (15, 16). The current study found that in DPN patients, SCs are subjected to high glucose (HG), which results in pathologically edematous cytoplasm, proliferation of cell bases, and reduction of neurotrophic factors, leading to mitochondrial dysfunction, localized oxidative damage, and the formation of immature cellular phenotypes, which in turn disrupts their support of neurons triggering neuropathy (4, 17). With more in-depth studies, HG was found to have a significant impact on the death regulation process of SCs.

Programmed cell death (PCD) is an active death process (e.g., apoptosis, autophagy, pyroptosis, ferroptosis, and necroptosis) that occurs after a cell receives a certain stimulus or signal in order to maintain the homeostasis of the internal environment (18), this process is important for the elimination of damaged cells, and the disruption of the process will result in the impairment of the function of normal cells and organs or even the inability to sustain the basic life (19). The effect of HG on this process in SCs may be an important part of the pathogenesis of DPN, which also suggests that intervening in the death of SCs by targeting and alleviating the disrupted PCDs pathway may serve as an effective way to treat DPN. Therefore, our narrative review highlights the current molecular mechanisms and understanding of HG damage to common PCDs in SCs, including apoptosis, autophagy, pyroptosis, ferroptosis, and necroptosis, to characterize the underlying mechanisms of diabetes-induced damage to SCs, highlight new insights, and provide perspectives on preventing as well as treating DPN by targeting the mode of death of SCs.

## SCs are an important link in the pathogenesis of DPN: the role of axons

**Figure 1:**
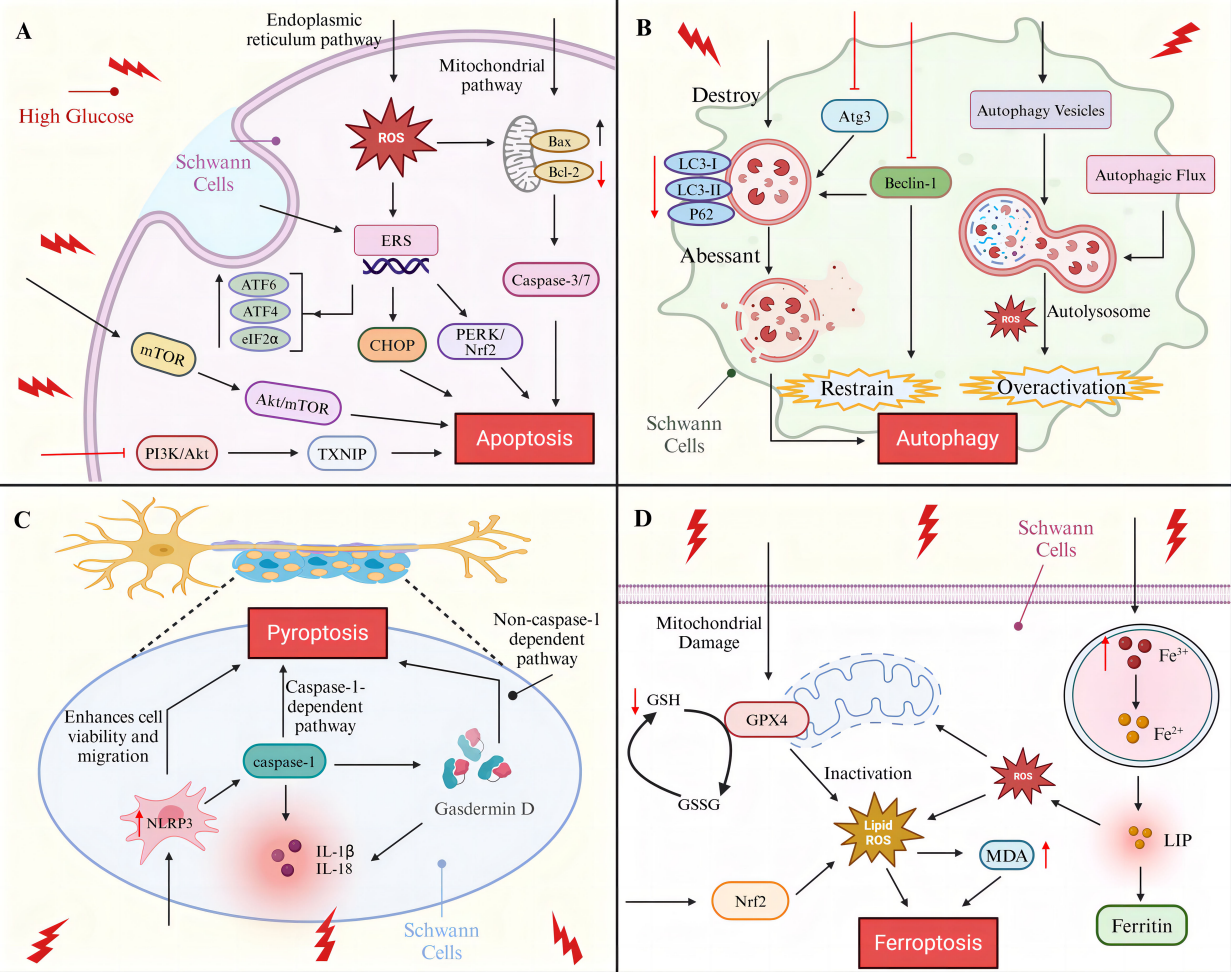
Programmed cell death of SCs in diabetic peripheral neuropathy. **(A)** SCs apoptosis pathway **(B)** SCs autophagy molecular pathway **(C)** SCs pyroptosis-associated signaling pathway **(D)** SCs ferroptosis mechanisms.

SCs encapsulate all axons in peripheral nerves and maintain the long-term functional integrity of axons (20), they have also been shown to provide trophic support to axons (21). Given their close association with axons, axonal damage due to abnormal function and metabolism of SCs is an important component of neuropathy. Increased polyol pathway flux is a recognized pathway for HG-induced DPN pathogenesis (22, 23). Aldose reductase (AR), the rate-limiting enzyme of the polyol pathway, is highly expressed by SCs in the peripheral nervous system, and overactivity of the polyol pathway in diabetic conditions, especially in SCs, leads to multiple metabolic abnormalities related to DPN (24). Overactivity of this pathway and altered biology of SCs may also be important for impaired axonal regeneration, one of the characteristics of the DPN (25). The lipid metabolism of SCs can transfer long-chain fatty acids (LCFA) to the mitochondria for β-oxidation to produce acetyl-coenzyme A, which enters the TCA cycle and eventually contributes to the production of OxPhos and ATP. In patients with type 2 diabetes mellitus, when there is an overload of the substrate, the remaining acetyl-coenzyme A is unable to enter the new round of β-oxidation and the TCA cycle, and is converted to acyl-carnitine. Carnitine accumulates in SCs and is eventually released into axons, causing axonal toxicity and nerve damage (26, 27). The maintenance of Ca^2+^ homeostasis plays an important role in axon function and integrity (28), whereas acyl-carnitine allow extracellular Ca^2+^ to enter axons, leading to abnormal axonal mitochondrial function and the onset of apoptosis (29, 30). Functionally, HG leads to the overproduction of advanced glycosylation end products (AGEs), and the structure and function of SCs can be altered by AGE-induced changes in key proteins, lipids, and nucleic acids, which in turn affect axons, leading to the development of neuropathy (31, 32). SCs mitochondrial function has been shown to be critical for maintaining axon survival, and studies have shown that HG is able to alter mitochondrial respiration, which may lead to SCs mitochondrial dysfunction (33, 34). In conclusion, the above process suggests that SCs may play a non-negligible role in the progression of DPN pathogenesis by acting axonal (**Figure 1**).

## Apoptosis

Apoptosis is the most prominent and widely studied mode of PCD during development (35), the process relies on the activation of a family of cysteine proteases to form an irreversible apoptotic pathway that is morphologically characterized by cell shrinkage or fragmentation, chromosome condensation, plasma membrane vesiculation, and DNA fragmentation (36, 37). The process of apoptosis is regulated by three major pathways: the mitochondrial apoptotic pathway, which is primarily controlled by the Bcl-2 family of proteins; the death receptor pathway, which is characterized by the binding of death receptors to ligands; and the endoplasmic reticulum (ER) apoptotic pathway, which consists of three apoptotic pathways activated by the UPR (IRE1α, PERK, and ATF6 pathways) (19). HG was identified as a crucial initiator of neuronal apoptosis, and HG was found to induce late neural tube apoptosis during gestation in mice (38). The administration of HG to adult gerbils subjected to forebrain ischemia results in the exacerbation of neuronal apoptosis (39). HG causes apoptosis in rat superior cervical ganglion neurons *in vitro* (40).

The apoptosis of SCs was successfully induced by HG. It was demonstrated that HG could cause the early activation of caspase-3/7 in SCs *in vitro* and *in vivo*, inducing caspase-dependent apoptosis in SCs (41). The results of an *in vivo* study demonstrated that the apoptosis rate of RSC96 cells treated with HG for 48 h was increased by 9.72-fold, confirming that high glucose also elevated the mitochondrial pathway apoptosis-related Bax/Bcl-2 and cleaved caspase-9/caspase-9 ratios, and suggested that knockdown of histone deacetylase (HDAC1) could effectively inhibit HG-promoted mitochondrial apoptosis (42). Endoplasmic reticulum stress (ERS) is an important pathway for apoptosis, and C/EBP homologous protein (CHOP) is the most important apoptotic factor induced by ERS (43), Wu et al. found that the ratio of ERS-activated pro-apoptotic protein CHOP to anti-apoptotic protein ORP150 increased under long-term HG conditions (after 16 weeks), leading to apoptosis (44). The RNA-dependent protein kinase-like ER kinase (PERK) pathway, one of the three apoptotic pathways of the endoplasmic reticulum, PERK/nuclear factor-E2-related factor 2 (Nrf2) play important roles in oxidative stress and ERS (45). It has been demonstrated that the PERK/Nrf2 pathway is activated in the DPN model, thereby promoting apoptosis through the up-regulation of CHOP expression. However, the intervention of traditional Chinese medicine TangLuoning has been shown to reduce the level of CHOP, thereby inhibiting ERS (46). In addition, Salem et al. found an increase in the relative expression of ERS pathway markers ATF6, PERK, ATF4, eIF2α, and CHOP in SCs in the HG environment as determined by protein blotting, suggesting that ERS active (47). Padilla et al. experimental study found that HG-conditioned culture increased lipotoxic damage in SCs and confirmed that this process first occurs by triggering ERS leading to apoptosis (48). In a study conducted by Li et al., it was discovered that excessive ERS in SCs under a HG environment was associated with the inhibition of PI3K/Akt/glycogen synthase kinase 3 beta (GSK3β) and ERK1/2 signaling pathways. Additionally, the administration of nerve growth factor (NGF) was observed to regulate neuronal growth and promote the restoration of DPN. Furthermore, the aforementioned study demonstrated that NGF administration inhibited ERS through the activation of the aforementioned pathways, thereby achieving a protective effect on SCs (49). Oxidative stress is another key pathway for HG-mediated apoptosis in SCs. As the main source and target of reactive oxygen species (ROS) (50), the function of mitochondria is affected by the content of ROS. Elevated ROS cause apoptosis by increasing the permeability of the outer mitochondrial membrane leading to the release of pro-apoptotic factors, such as cytochrome c, caspase, etc. (51–53). The study found that the level of ROS in HG-treated SCs was significantly higher than that of the control by 1.54 times, suggesting that HG may lead to SCs apoptosis by inducing mitochondrial oxidative stress damage (54), whereas, salvianolic acid B (SalB) and geranylgeranyl exhibited inhibitory effects on HG-induced oxidative stress and mitochondrial depolarization in a dose-dependent manner, thereby protecting SCs from apoptosis (55, 56). More studies have shown that long-term HG similarly leads to ROS accumulation in the endoplasmic reticulum and induces ERS (57), interestingly ERS has the potential to contribute to elevated mitochondrial ROS levels, an undesirable interaction that accelerates cell death (46). CXC motif chemokines are involved in neuronal injury, CXC motif chemokine ligand 2 (CXCL2) expression is significantly increased in HG-induced SCs, CXCL2 knockdown increases SCs viability and reduces cleaved caspase 3/9 expression and associated apoptosis (58).

In addition to the above regulatory pathways, the role of HG on signaling pathways is critical in inducing apoptosis in SCs. One of the downstream targets of AMPK, rapamycin (mTOR), plays a role in the apoptosis and autophagy of SCs in the development of DPN disease (59). A study conducted on RSC96 cells revealed that the Akt/mTOR signaling pathway was inhibited and apoptosis and autophagy were increased under HG conditions. Furthermore, the inhibition of the mTOR kinase protein complex mTORC1 in RSC96 cells promoted apoptosis by silencing PARTOR or RICTOR. Additionally, animal experiments yielded similar results, demonstrating that in the sciatic nerves of diabetic mice, the phosphorylation of mTOR decreased, and the apoptosis of Schwann cells increased in diabetic mice (60–62). PI3K/Akt signaling is strongly associated with the prevention of apoptosis (63), thioredoxin interacting protein (TXNIP) plays an important role in inflammatory response, apoptosis (64), Zhang et al. found that HG promotion of SCs apoptosis was associated with TXNIP upregulation and was shown to be achieved by inhibiting the PI3K/Akt pathway (65), whereas artesunate (ART) was shown to inhibit SCs apoptosis and promote SCs viability by acting on the PI3K/AKT/mTOR signaling pathway in ex vivo experiments (66). Extracellular signal-regulated kinase (EPK) as a multifunctional protein kinase, activation of the EPK pathway has been shown to be involved in apoptosis in glial cells (67, 68). Liu et al. found that the decrease in SCs viability and the increase in SCs apoptosis under HG conditions paralleled the increase in ERK phosphorylation levels, suggesting that EPK activation may play a role in HG-induced SCs apoptosis (69), but the exact mechanism remains to be further clarified.

Additionally, dyslipidemia and insulin deficiency are significant predisposing factors for the development of DPN. Diabetes mellitus complicated by dyslipidemia with elevated oxidized low-density lipoprotein (oxLDL) levels affects the progression of DPN. *In vitro* experiments have demonstrated that the intervention of HG with oxLDL leads to the activation of the Caspase-3 pathway by inducing the overactivation of Toll-like receptor 4 (TLR4) signaling in SCs, which promotes the development of apoptosis (70). Insulin-like growth factor-1 (IGF-1) is a polypeptide protein that exhibits molecular similarity to insulin and is capable of exerting a range of metabolic effects analogous to those of insulin (71). Previous studies have demonstrated that IGF-1 plays a regulatory role in the viability of developing SCs and has been shown to protect SCs from apoptosis through caspase activation of PI3-K signaling, which is located upstream. Subsequent studies have found that high levels of IGF-1 (10 nm) are protective, whereas cells treated with 0.3 nm IGF-I succumbed to cellular apoptosis (41, 72, 73). A recent clinical study has confirmed that there is an association between IGF-1 deficiency and DPN. Furthermore, the study has indicated that there is a potential risk factor for DPN in patients with lower levels of IGF-1. The findings demonstrate that patients with DPN have significantly lower levels of IGF-1 compared to patients without DPN (74). Moreover, an *in vivo* experiment demonstrated that islet transplantation effectively reversed sciatic nerve injury in diabetic mice. Additionally, islet cells exhibited a protective effect on RSC96 cells under HG conditions through the activation of the mTOR/S6 kinase 1 (S6K1) pathway (75). It can be reasonably deduced that the investigation of pertinent therapeutic modalities that target lipids and insulin is crucial to enhance the progression of DPN lesions.

## Autophagy

Autophagy is an important intracellular degradation and recycling process that renews and generates new building blocks and energy for the cell by recycling and degrading intracellular components and damaged organelles, thus acting as a dynamic recycling system (76, 77). Normal autophagy sessions in the nervous system facilitate the maintenance of neuronal integrity as well as synaptic plasticity (78), autophagy also promotes recovery and regeneration of peripheral nerve function by improving myelin and axonal regeneration (79). Not only that, but studies in recent years have shown that autophagy is involved in the formation of neuropathic pain, and that insufficient autophagic activity may lead to neuropathic pain development (80). The autophagy mechanism of SCs has even been shown to favor scar reduction and myelin formation, which is important in preventing or delaying the onset and chronicity of neuropathic pain and neuropathy (81, 82). It has even been suggested that autophagy dysfunction in SCs may be the root cause of DPN (83). Therefore, an in-depth understanding of the disruption of the autophagic mechanism of SCs by HG is extremely important for DPN as well as neuropathic pain.

Autophagy in SCs was shown to be inhibited in HG environments, microtubule-associated protein light chain 3 (LC3) was a key readout for cellular autophagy levels (84), Du et al. found that autophagy markers LC3-II/LC3-I and P62 were significantly reduced in rat SCs in HG environment compared to normal glucose-treated cells (85). Zhang et al. improved autophagy inhibition in SCs by knocking down TXNIP in HG-cultured SCs, effectively preventing the HG-induced downregulation of LC3-II/LC3-I ratio (65). Qu et al. not only found that LC3 protein expression was down-regulated in SCs in the HG environment, but also observed the appearance of morphologically and structurally abnormal autophagosomes in the cells, suggesting that autophagy is disrupted in SCs (86). Beclin-1 as an important gene in the autophagic process, Beclin-1/VPS34 controls phagocytosis in response to stress signaling pathways in the ER and other membranes (87), Yuan et al. experimentally found that HG reduced the expression of beclin-1 and autophagy-related gene Atg3 in SCs, nevertheless, strychnine treatment via the AMPK pathway negated the correlation and augmented the expression of the SCs autophagosome marker LC3-II, effectively inhibiting autophagy occurrence (88). Similarly, Gao et al. demonstrated that rats with DPN induced by HG exhibited reduced Beclin-1 and LC3-II/LC3-I ratios in SCs. They proposed that translocator protein (TSPO) agonists exert a therapeutic effect on DPN through the regulation of autophagy in SCs (89). In addition, oxidative stress is also involved in the autophagic process, and the HG-induced increase in ROS levels may contribute to autophagic dysfunction in SCs (83). More interestingly, it was found that there may be over-activation of autophagy in SCs by HG, Wei et al. observed abundant autophagic vesicles and enhanced autophagic flux in SCs from the HG group (90), autophagy hyperactivity was also observed in the streptozotocin (STZ)-induced rat model of DPN in Wang et al., these effects were also found to potentially increase apoptosis in SCs and demyelination of sciatic nerves in rats with DPN (91). A recent *in vivo* and ex vivo study revealed that phosphorylated mTOR and p62 expression was markedly elevated in SCs treated with HG, indicating that HG may facilitate autophagy by influencing mitochondrial function, whereas the combination of cannabidiol (CBD) and Beta-Caryophyllene (BC) pretreatment demonstrated a substantial protective effect against mitochondrial autophagy in SCs (92).

## Pyroptosis

Neuroinflammation is emerging as a pivotal factor in diabetes-induced peripheral neuropathy. Chronic HG has been linked to advanced glycation end products AGEs formation, which in turn activates intracellular signaling and promotes the expression of pro-inflammatory transcription factors and the release of various inflammatory cytokines (93). The results of animal experiments indicated that elevated serum TNFα expression in DPN rats and elevated sciatic nerve IL-6 and IL-1β levels in diabetic rats were associated with neuropathy, this suggests that inflammatory factors may play a role in the pathogenesis of neuropathy (94, 95). Moreover, several clinical studies have identified elevated levels of numerous inflammatory markers, including CRP, TNF-α, IL-1RA, sTNFR1, and sTNFR2, along with elevated serum NF-κB levels, in patients with DPN compared to patients with T2DM without neuropathy, suggesting that patients with DPN exhibit a more pronounced inflammatory response (96–98). More significantly, elevated plasma levels of pro-inflammatory factors have been demonstrated to play a pivotal role in predicting the incidence of DPN. Of these factors, the IL-6 inflammatory factor has been shown to be particularly relevant, exhibiting a distinct difference between painful and painless DPN (99, 100). The presented evidence indicates a strong correlation between inflammatory processes and DPN.

Pyroptosis is a cleavage PCD triggered by inflammatory vesicles (101), it is dependent on members of the Gasdermin protein family to form plasma membrane pores and shares DNA breakage, nuclear condensation and caspase-dependent morphological alterations with apoptosis (102). The process of pyroptosis mainly involves the caspase-1-dependent pathway (classical pathway) and the caspase-1-independent pathway (non-classical pathway), and is closely related to the secretion and activation of the pro-inflammatory cytokines interleukin-1β (IL-1β) and IL-18, which play an important role in inflammatory response and immune defense through the release of inflammatory mediators and the recruitment of immune cells (103–105). Pyroptosis has been shown to play a crucial role in the development of diabetes and its complications (106, 107). NLRP3 inflammasome is a cytoplasmic multiprotein complex that is commonly aberrantly activated in inflammatory diseases such as diabetes to induce pyroptosis (108). Cheng et al. observed a significant increase in the protein expression of NLRP3 and Gasdermin D in SCs under HG (25 mM) environment, which promoted the development of SCs pyroptosis (109);Wang et al. found that taurine deoxycholic acid (TUDCA) effectively inhibited pyroptosis in HG-stimulated SCs by targeting NLRP3 expression to enhance cell viability and migration (110). Immunofluorescence staining and quantitative RT-PCR confirmed that pyroptosis-related proteins cleaved-GSDMD, NLRP3, caspase-1, IL-1β, and IL-18 were significantly enhanced in HG-stimulated SCs (106). These findings strongly suggest that HG induces the onset of SCs pyroptosis and that pyroptotic SCs inhibit neuronal function and promote an inflammatory response (111), thus inhibiting pyroptosis in SCs has implications for neurons. Furthermore, NF-κB plays a pivotal role in the pathogenesis of inflammatory neuropathies. It is a crucial mediator of immune, inflammatory, and apoptotic responses, which are initiated by the activation of the RAGE pathway. A study of 45 diabetic patients with peroneal nerve biopsies revealed that RAGE was upregulated in SCs, this suggests that RAGE may be a key regulator of apoptosis or pyroptosis in SCs. However, the precise mechanism by which RAGE exerts its effects remains to be elucidated (112, 113). A recent study demonstrated that the Ras-related protein Rab32 induces significant oxidative stress by disrupting the mitochondria of SCs, thereby exacerbating SCs pyroptosis in peripheral nerve injury, it would be beneficial to investigate whether Rab32 plays a role in the death process of SCs in DPN (114).

## Ferroptosis

Diabetes mellitus as a lifelong metabolic disease due to the presence of disturbances in cellular metabolism leading to ferroptosis and ferritin autophagy (115). Ferroptosis is an iron-dependent regulated necrosis that is considered a byproduct of cellular metabolism because of its close association with oxygen, iron, and lipid metabolism (116), it is dependent on the metabolites reactive oxygen species (ROS), phospholipids containing polyunsaturated fatty acid chains (PUFA-PL), and the transition metal iron, and exhibits biochemical features of iron accumulation and unrestricted lipid peroxidation, as well as morphologic alterations such as loss of plasma membrane integrity, mitochondrial abnormalities, cytoplasmic swelling, and moderate chromatin condensation (117, 118). Briefly, Fe3+ is deoxygenated to catalytically active Fe2 + and released into the cytoplasmic labile iron pool (LIP) to be utilized or stored as ferritin in the cell. when the intracellular Fe2+ overload exceeds the caching capacity of ferritin, and intracellular free iron generates ROS through the fenton reaction, and glutathione (GSH), one of the critically important antioxidants in cells, is catalyzed by peroxidase to oxidized glutathione (GSSG) during lipid peroxidation, and inhibit lipid peroxidation-induced ferroptosis (19). The typical ferroptosis mechanism is mediated by glutathione peroxidase 4 (GPX4), and inactivation or knockdown of GPX4 leads to the onset of ferroptosis (119). It was confirmed that serum GPX4 and GSH were significantly reduced and lipid peroxides MDA were significantly elevated in DPN patients, suggesting that ferroptosis levels were elevated in DPN patients, and it was found that HG induced ferroptosis in SCs through the inhibition of a pathway directly downstream of ROS (NRF2 signaling pathway) (120). Hu et al. found that and honokiol reduced HG-induced ferroptosis and SCs mitochondrial dysfunction by acting on the AMPK/SIRT1/PGC-1α axis and downstream gene expression profiles (121). Even though some studies have shown a crossover between the underlying pathologic mechanisms of DPN and ferroptosis (115), and the above experiments have confirmed that HG exerts an effect on ferroptosis in SCs, However, overall more research is needed on the mechanisms of ferroptosis in DPN. For example, a recent report indicates that arachidonic acid 15-lipoxygenase (ALOX15), which is highly correlated with ferroptosis, is highly expressed in human Schwann cells, and also has an important role in functional changes in the DPN, as well as in demyelination, in light of these findings, it would be beneficial to investigate whether HG influences ferroptosis in SCs by modulating ALOX15 activity (122).

## Necroptosis

Necroptosis is a regulated mode of cell death induced by environmental stimuli, which is mainly regulated by the TNF-α receptor system and ultimately directly activates and regulates the inflammatory response through the loss of membrane integrity and the efflux of intracellular content (123, 124). MLKL, a key mediator of necroptosis, is phosphorylated by RIP3 kinase and disrupts plasma membrane integrity leading to necrosis (125). Guo et al. demonstrated that MLKL mediates DPN demyelination by *in vivo* and *in vitro* observations and found that MLKL levels were increased in the gastrocnemius muscle of STZ-induced diabetic mice and that MLKL signaling was prominent in the myelin sheaths of patients with DPN (126), Demyelination of SCs is associated with a “sock and glove” pattern in patients with DPN, and the process described above involves MLKL-mediated damage to SCs, which may be the result of necroptosis in SCs (127), but Guo et al. did not detect necroptotic apoptotic MLKL phosphorylation in diabetic patients (126). The component nuclear pore protein Seh1 plays a role in protecting the homeostasis of SCs by maintaining genomic integrity, and it has been shown that interactions following Seh1 ablation trigger ZBP1-dependent necroptosis in SCs leading to peripheral neuropathy (128), however, whether HG has an effect on this link is not clear.

## Cross-targeting cell death patterns and therapeutic perspectives

Although each of these pathways has specific mechanisms and outcomes, different cell death regulatory pathways crosstalk with each other. Crosstalk between apoptosis and autophagy is regulated by multiple factors such as Ca2+ signaling, Bcl-2 and caspase-3/8 factors, and oxidative stress (129–131), Beclin-1 is a direct bridge between the two signaling pathways and plays an important role in the communication between autophagy and apoptosis (132). The calcium mimetic cinacalcet has been shown to regulate Ca2+ levels and increase Beclin-1 and Bcl-2/Bax expression in SCs in the diabetic sciatic nerve, and to play an important role in the restoration and amelioration of DPN through the regulation of apoptosis and autophagy (133). In addition to its important role in apoptosis, ER is likewise at the crossroads of two pathways, apoptosis and autophagy, in the diabetes process (134), Melatonin (MEL) as an antioxidant and anti-inflammatory agent was found to play a role in the ER major stress response (135). Negi et al. found that MEL can regulate neuroinflammation through activation of the Nrf2 pathway (136), another experimental and clinical study confirmed the protective effect of MEL on glial cells and its beneficial effect on DPN, but the specific therapeutic regimen of administration needs to be further clarified (137). Pro-inflammatory cytokines similarly link autophagy to apoptosis and have been shown to be associated with neuropathic pain (138), This phenomenon suggests that we may be able to further explore whether there are pro-inflammatory cytokine-related drugs to treat neuropathic pain in DPN patients by improving the apoptosis and autophagy link.

The establishment of the concept of PANoptosis (which refers to the inflammatory PCD pathway regulated by the PANoptosome complex, encompassing pyroptosis, apoptosis, and necroptosis, and which cannot be explained by one or the other alone) strongly suggests that there is a broad crosstalk between apoptosis, pyroptosis, and necroptosis (139, 140). NLRP3 inflammasome is important for the crosstalk between the three, CASP8-NLRP3 can connect pyroptosis with apoptosis; MLKL and macrophage potassium efflux-mediated activation of NLRP3 inflammasome crosstalk pyroptosis with necroptosis (summarized in (141)). ZBP1-NLRP3 inflammatory vesicles were even shown to be key components of PANoptosis (142). In addition, NLRP3 inflammatory vesicles play a role in peripheral neuropathic pain, which has been highlighted as having the potential to be a therapeutic target for neuropathic pain (143). Taurine deoxycholic acid (TUDCA), loganin, and CXCL2 blocking therapy all have the effect of modulating NLRP3, and all three have also been shown to improve SCs mortality in the HG environment (58, 109, 110). Caspases are dominant in apoptosis and pyroptosis, apoptotic Caspase-3 and 8 mediate pyroptosis by cleaving Gasdermin and thus Caspase-8 is even more important to maintain the balance between apoptosis and necroptosis (141, 144), thus, strict adjustment of the level of caspase in SCs is important to improve PCD sessions.

The crosstalk between autophagy and ferroptosis at the molecular level was revealed by Hou et al. They demonstrated that autophagy triggers ferroptosis through degradation of ferritin, and found that autophagy mediated by autophagy-associated genes Atg5 and Atg7 likewise led to ferroptosis (145). The GPX4 essential cofactor GSH not only plays an important role in ferroptosis, but GSH depletion also induces a significant increase in autophagy (146), whereas erythropoietin (EPO) was able to increase the level of total GSH, reduce ROS levels, and improve cell viability in SCs in the HG environment (147). The human tumor suppressor protein p53 not only regulates autophagy in a dual manner, nuclear p53 is revealed to stimulate ferroptosis in a transcription-dependent manner (148, 149), Li et al. also showed that p53 may be a key molecule in the ferroptosis - apoptosis crosstalk (150), and as previous studies have long found that p53 is critical for neuronal death (151, 152), we believe that p53 may be a potential therapeutic target. Erastin acts as a ferroptosis activator, which not only induces ferroptosis, but also causes apoptosis to occur (153). The combined action of erastin and the apoptotic agent TRAIL was shown to mediate apoptosis via activation of caspases (154), suggesting a possible mechanism of interaction between ferroptosis and apoptosis. Furthermore, ER and mitochondria are key organelles in PCD, Lee et al. found that ferroptosis-induced ERS crosstalks ferroptosis with apoptosis by increasing the expression of the pro-apoptotic molecule PUMA (155). The Bax-mediated mitochondrial pathway not only affects apoptosis but also mediates ferroptosis by acting upstream of SLC7A11 and GPX4 (156). In addition, Nrf2, as an important participant in redox and inflammatory responses, not only mediates the process of apoptosis, but also participates in the regulation of autophagy and ferroptosis during disease therapy (157). The expression of Nrf2 has been shown to promote SCs-mediated neurological recovery in DPN (158), whereas salvianolic acid A, paeoniflorin, and the traditional Chinese medicine Tangluoning were all found to have a modulatory effect on Nrf2 expression in SCs in the HG environment (46, 159, 160).

## Concluding remarks

**Figure 2:**
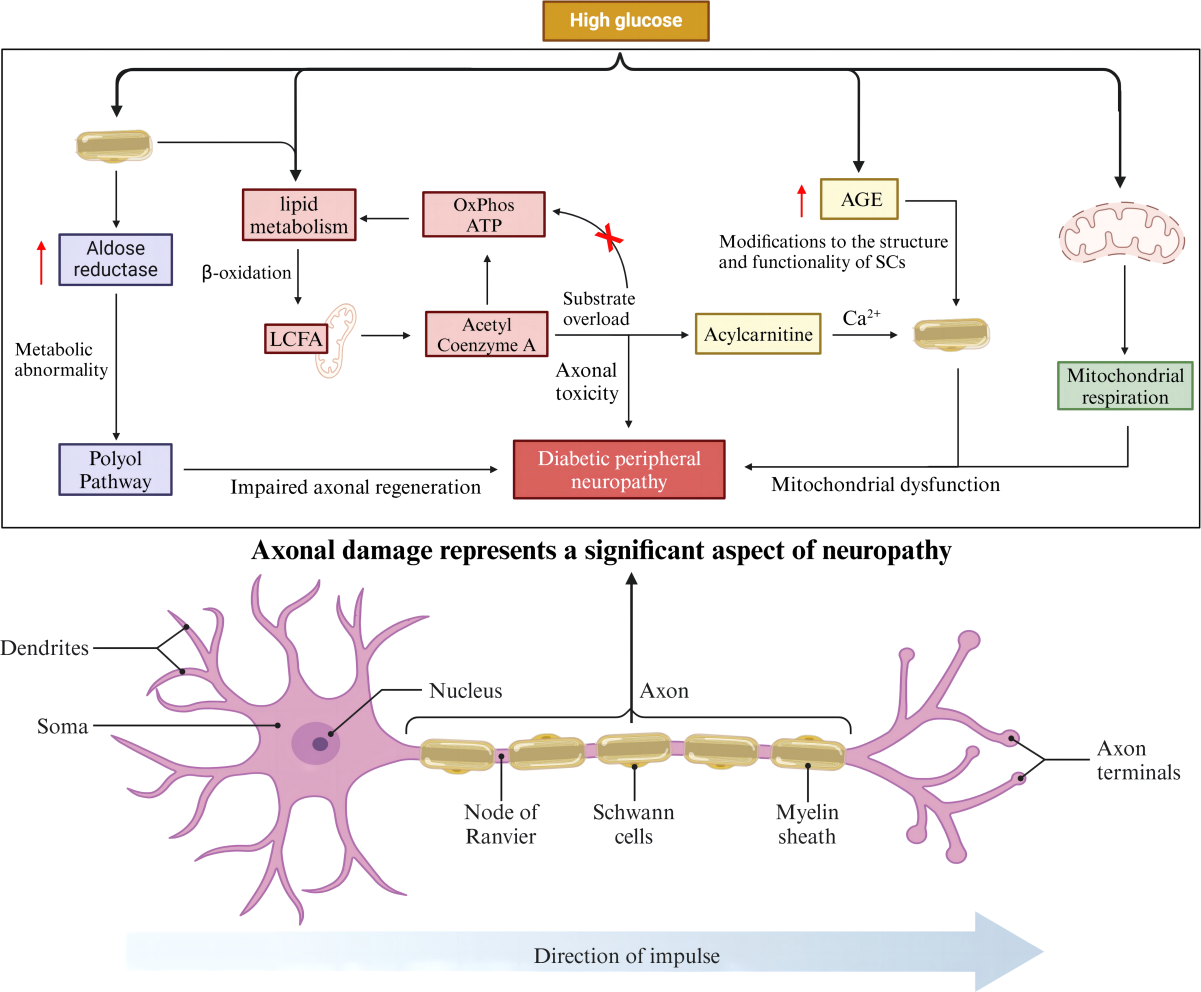
SCs-associated axonal injury.

SCs have an extremely important role in the peripheral nervous system, and the disruption of the death process of SCs by HG is an important part of DPN occurrence and development. In this review, we discuss the specific molecular mechanisms by which several forms of PCD in SCs are affected (**Figure 2**), and exemplifies some of the drugs that ameliorate the death of SCs through the corresponding linkages (**Table 1**). For example, HG induces apoptosis in SCs through multiple pathways involving disruption of mitochondrial function, endoplasmic reticulum stress, as well as oxidative stress and signaling pathways; inhibition or over-activation of autophagy is achieved by decreasing the expression of LC3-II/LC3-I, beclin-1, and autophagy genes in SCs, or by increasing autophagy fluxes. Among them, HG-induced inflammatory response is the culprit for the pyroptosis of SCs, and the aberrant activation of NLRP3 inflammatory vesicles is the key link for the induction of pyroptosis and PANoptosis, which suggests that targeting the NLRP3 inflammatory vesicles is a reliable therapeutic pathway. Unfortunately, although a few studies have observed the effects of HG on ferroptosis and necroptosis in SCs, the molecular mechanisms involved are relatively homogeneous and more and deeper mechanistic explorations are lacking, thus these two pathways need to be further explored, especially necroptosis. We conclude by describing the relevant targets of crosstalk of these PCDs, pointing out that some of the drugs targeting the targets are effective in mitigating DPN progression and neuroinflammation, but there is still a therapeutic void for important targets like p53.

**Table 1:** Targets related to cell death in SCs.

Programmed cell death	Regulatory pathway	Targets and signaling pathways	Active substance	References
Apoptosis	Mitochondrial Apoptotic Pathway	Caspase-3/7	—	(41)
		Bax/Bcl-2, caspase-9/caspase-9 ratios	HDAC1	(42)
	Endoplasmic Reticulum Stress	CHOP	TangLuoning	(46)
		PI3K/Akt/GSK3β, ERK1/2	NGF	(49)
		PERK, PERK/Nrf2, ATF6, ATF4, eIF2α	—	(43, 44, 47)
	Oxidative Stress	Cytochrome c, caspase	—	(51–53)
		ROS, Mitochondrial damage	SalB, geranylgeranyl	(55, 56)
	Other paths	Akt/mTOR, mTORC1	—	(60–62)
		TXNIP, PI3K/AKT/mTOR	artesunate	(66)
		Caspase, PI3-K	IGF-1	(41, 72, 73)
		EPK, TLR4, Caspase-3	—	(67, 68, 70),
Autophagy	Inhibitory	beclin-1, Atg3, LC3-II	strychnine, Translocator agonists	(88, 89)
		LC3, LC3-II/LC3-I, P62, TXNIP	—	(85, 65),
	Over-activation	mTOR, p62	CBD, BC	(92)
Pyroptosis	Classical pathway	NLRP3	TUDCA	(110)
		Gasdermin D, caspase-1, IL-1β, IL-18	—	(106)
	Other paths	RAGE, Rab32	—	(112, 113, 114)
Ferroptosis	—	AMPK/SIRT1/PGC-1α	honokiol	(121)
	—	GPX4, GSH, Nrf2, ALOX15	—	(120, 122)
necroptosis	—	MLKL, RIP3, Seh1	—	(125, 128)

It is worth noting that despite significant progress in potential research on the cellular and molecular levels of SCs death, nevertheless, apart from a few encouraging preclinical studies, there is a dearth of clinical studies targeting the Schwann cell pathway for the treatment of DPN. The majority of the promising therapeutic agents currently available remain in ex vivo studies lacking robust clinical validation. Consequently, there is an imperative need for broader and more comprehensive clinical exploration as a means of combating DPN. In addition, although SCs autophagy and NLRP3 were found to be possible therapeutic directions for neuropathic pain, the specific effective mechanisms in DPN need to be further clarified. In conclusion, we believe that there is an urgent need to explore DPN therapies targeting different forms of PCDs in HG-induced SCs and the molecular mechanisms that crosstalk with each other, and, despite the many challenges, we strongly believe that thorough experimental and clinical studies can make the protection of SCs PCDs an important target for the intervention and treatment of DPN.
